# A longitudinal application of the Actor Partner Interdependence Model extended Mediations to the health effects of dyadic support

**DOI:** 10.1371/journal.pone.0254716

**Published:** 2021-07-19

**Authors:** Serena Petrocchi, Chiara Filipponi, Peter J. Schulz

**Affiliations:** 1 Faculty of Biomedical Sciences, Università della Svizzera italiana, Lugano, Switzerland; 2 Lab of Applied Psychology, Department of History, Society, and Human Studies, University of Salento, Lecce, Italy; 3 Faculty of Communication, Culture and Society, Institute of Communication and Health, Università della Svizzera italiana, Lugano, Switzerland; 4 Department of Oncology and Hemato-Oncology, University of Milan, Milan, Italy; 5 Applied Research Division for Cognitive and Psychological Science, IEO European Institute of Oncology IRCCS, Milan, Italy; University of Bologna, ITALY

## Abstract

Supportive communicative experiences within close relationships, such as dyadic support, have a protective effect on individuals’ health and emotional well-being. However, little is known about how partners interact in determining their own and others’ health or the mechanisms through which dyadic support influences physical health. We addressed those gaps by studying 1088 romantic couples from three consecutive years (T1, T2, T3; Swiss Household Panel). The study applied a data analysis strategy called Actor Partner Interdependence Model extended Mediation, which allows for mediation processes while considering the interdependence, or non-independence, of data coming from partners. Results showed that dyadic support was positively associated with perceived health over two years through the mediation of optimistic attitudes and depressive mood, both for person and partner effects. The present study demonstrates the interplay between the dyadic process and personality dispositions in maintaining good health.

## Introduction

According to the Social Penetration Theory [1], the development and maintenance of a close relationship is guaranteed by partners’ willingness to exchange different types of information. Self-disclosure increases intimacy and perception of the quality of support received by the partner [2]. Social Penetration Theory has been applied in different contexts [3], including romantic relationships, in which couples support each other in different ways (e.g., sharing information, giving advice, or providing emotional support to the other). Dyadic support, the support shared between two individuals in a close relationship, buffers the negative effect of stress and adversity on health [4, 5]. The mechanisms involved comprise immune system functioning [6], cortisol response to stress [7–9], and cardiovascular activity [10].

However, dyadic support exerts its effects on an individual’s health even in the absence of adversity [11–13]. Supportive communication within close relationships, such as between romantic partners, exerts its effect on health [14] even during non-stressful daily events [15, 16]. The present study places itself in this field of research of supportive communication processes between romantic partners, exploring two mechanisms explaining the longitudinal effect that dyadic support has on the partners’ health. For this purpose, the present research applied a model of analysis called the Actor-Partner Interdependence Model extended Mediation (APIMeM) [17–19], which considers the source of the interdependence of the dyad. Then, the theoretical basis for the APIMeM application is explained, and then the principles underlining the specific hypotheses of the present study are presented.

### The interdependence or non-independence of partners

According to the Interdependence Theory [20, 21], close relationships are characterized by strong interdependence, defined as the extent to which intimate partners influence each other. In other words, two scores of the same variable measured on partners of a relationship are more similar to (or different from) scores of individuals who are not in relationship. This concept is called interdependence (or non-independence) [22]. For example, research demonstrated that partners in a romantic relationship show similarity in their physiological response within a day [23] and during conflicts [24].

Like many other interpersonal phenomena [25–27], dyadic support requires considering the influential reciprocal processes and co-regulation of responses between partners. Data emerging from those processes necessitate a specific set of analysis set that considers its non-independence [22]. Among the statistical techniques applied for interpersonal phenomena, the Actor-Partner Interdependence Model (APIM) [28] and the APIMeM [17–19] are the two most prominent [22, 29].

The APIM and the APIMeM take into account the non-independence of the data coming from partners of a couple. The APIM, the most basic model, distinguishes between actor and partner effects. The actor effect is the variation in an outcome variable Y, measured on partner A, and determined by partner A’s characteristics. The partner effect is the variation in an outcome variable Y, measured on partner A, and determined by partner B’s characteristics. In each APIM estimation, researchers can calculate two actor effects (from partner A to A and from partner B to B) and two partner effects (from partner A to B and from partner B to A). How a person’s independent variable is associated with his/her score on the dependent variable is explained by the actor effect. In contrast, the partner effect explains how a person’s independent variable is associated with the score of his/her partner’s dependent variable, allowing the exploration of partners’ interdependency. Significant partner effects are evidence that the two persons are part of an interdependent system [22].

To reveal possible explanatory mechanisms underlining the relationship between the independent variable and the outcome of dyadic data, Ledermann and colleagues [19] have extended the APIM estimation to mediation analysis. The APIMeM allows for the effects of one or more mediator variables between the presumed causal agent X and an outcome Y to be measured on both members of a dyad. With this model, it is possible to estimate the direct and indirect effects of X on Y through one or more mediator (M) when M is measured for both members of the dyad. Essentially, four different effects could be estimated [30, 31]: two actor effects (i.e., one for husband and one for wife) and two partner effects (i.e., one for husband and one for wife). In this case, the indirect effects are of central importance, as they quantify and convey information about the mechanism by which X is connected to Y, and in line with this model, the mechanisms could operate through the person’s own M, the partner’s M, or both simultaneously. The present research applied the APIMeM to measure the effect of the dyadic support on partners’ health directly, and by the mediation of depression mood and optimistic attitude.

### Depression mood and optimistic attitude as mechanisms

A considerable amount of research demonstrated that dyadic support shared in close relationships affect partners’ mental health [4, 32–36]. Similarly, dyadic support is found to be directly associated with physical health [36–41]. The aforementioned evidence has proven the positive direct association between dyadic support and health; however, these studies have several limitations. First, they have a cross-sectional design and therefore lack longitudinal evidence. Secondly, the studies did not consider both members of the dyad simultaneously and did not take into account their interdependence. The present study aimed to fill those gaps, and the first hypothesis was as follows:

*Hypothesis 1 (HP1)*: *Perceived dyadic support exerts a positive influence on physical health over two years (i*.*e*., *direct actor effects)*. *In particular*, *the husband’s perception of dyadic support (i*.*e*., *partner A) received from the wife (i*.*e*., *partner B) influences his physical health (HP1a)*, *and the wife’s perception of dyadic support received from the husband influences her physical health (HP1b)*.

Other evidence [37–41] suggested that dyadic support may indirectly help partners maintain physical health. Pietromonaco and Collins [13] suggested that dyadic support could be linked to health through the mediation of several psychosocial, biological, and behavioral mediators. Among the psychological mediators, the authors suggested that the support received by the partner may minimize negative emotional responses that compromise health [42] and, at the same time, foster positive emotional responses that protect health [43]. A review of Blazer [44] revealed that dyadic support and dispositional optimism are protective factors for health, while the depressive mood is a risk factor. Moreover, other researchers [45–48] confirmed the association between optimism and health.

Dyadic support may help partners reach a well-adjusted affective state of mind, sustaining the emotional regulation processes and the self-regulatory resources [12] that may ultimately lead to better health behaviors (e.g., diet, exercise) which enhance overall health. Based on the principles mentioned above, the present research aimed to explore two mechanisms responsible for the effect of dyadic support on an individual’s health within close relationships between heterosexual partners. The second hypothesis is as follows:

*Hypothesis 2 (HP2)*: *Perceived dyadic support exerts a positive influence on physical health through depressive mood and optimistic attitudes over two years (i*.*e*., *indirect actor effects)*. *The husband’s perception of dyadic support (partner A) received from the wife (partner B) influences his negative mood and optimistic attitudes*, *which are associated with his perceived health (HP2a)*. *Similarly*, *the wife’s perception of dyadic support received from the husband influences her negative mood and optimistic attitudes*, *which are associated with her perceived health (HP2b)*.

Two final research questions were explored:

*Research Question 1 (RQ1)*: *Is there an influence on partner A’s perception of the dyadic support received by B on B’s physical health (i*.*e*., *direct partner effect); and*,

*Research Question 2 (RQ2)*: *Is there an influence on partner A’s perception of dyadic support received by B on B’s physical health through the mediation of depressive mood and optimistic attitudes (i*.*e*., *indirect partner effect)*?

**Fig 1:**
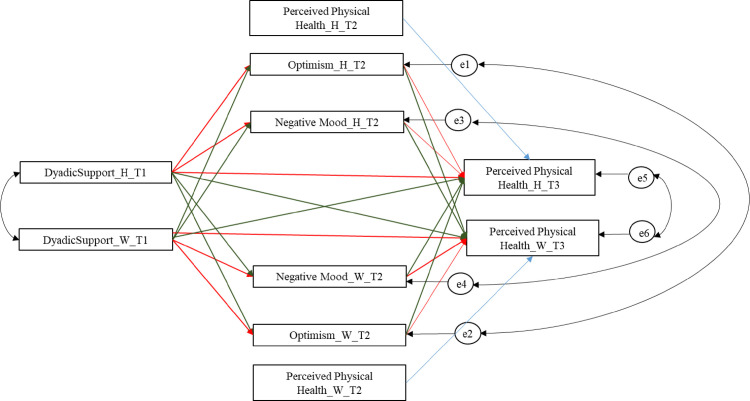
Theoretical model tested. *Note*: H = husband; W = wife. Dyadic support stands for all the predictor variables (emotional and practical support). Red lines indicate actor effects; green lines indicate partner effects. Data Source: Swiss Household Panel (SHP).

Fig 1 shows the full model estimated.

## Materials and methods

### Study participants

Data came from the Swiss Household Panel (SHP) [49], a large annual household panel study based on a random sample conducted in Switzerland since 1999. At present, the SHP [50] is comprised of three cohorts: the SHP_I (5,074 households and 7,799 individuals interviewed from 1999), the SHP_II (2,538 households and 3,654 individuals interviewed from 2004), and the SHP_III (3,989 households and 6,090 individuals interviewed from 2013 to present). The data collection followed the ethical standards defined by the Declaration of Helsinki.

We included data of the heterosexual couples of three waves: 2013 (wave 15), 2014 (wave 16), and 2015 (wave 17), henceforth T1, T2, and T3. The initial sample was composed of 5358 individuals (n = 2679 dyads); 3182 were excluded from the analyses because more than 10% of their data from variables considered were missing completely at Random (MCAR): *χ*^2^ = 12,02, df = 7, p = .10. The analytical sample was composed of 2176 individuals (age range 24–89, Mhusbands = 58 years, SD = 11.2; Mwives = 55 years, SD = 11.4; N = 1088 dyads). For the analytical sample, the mean of the years of the highest educational degree based on the ISCED-classification scheme [51] was 14.72 for men (sd = 3; range 8–21) and 13.27 for women (sd = 2.9; range 8–21). Sixty-eight percent of the men were employed, 32% retired, and 1% unemployed, while 58% of the women were employed, 25% retired, and 17% unemployed. The distribution of the children belonging to the family nucleus was as follows: 12% of the couples did not have any kids, 13% had one, 45% had two, 22% had three, 5% had four, 1% had five, and 4% had six.

T-tests comparing individuals in the initial sample and the analytical sample are shown in Table 1. Partners in the analytical sample were older than partners in the initial sample and slightly more educated. They reported higher dyadic support and optimistic attitudes, less depressive mood, and greater perceived health than participants in the initial sample.

**Table 1:** Independent sample t-tests comparing the initial and the analytical sample.

	Analytical	Initial	t-test(df)
	sample	sample	
	M(ds)	M(ds)	
Age			
Husbands	58.41 (11.22)	52.35 (16.17)	t(2676.68) = -11.44***
Wives	55.39 (11.49)	49.74 (15.73)	t(2666.6) = -10.74***
Years of Education			
Husbands	14.72 (2.99)	14.15 (3.03)	t(2676) = -1.89*
Wives	13.27 (2.90)	13.06 (2.90)	t(2676) = -4.78***
Emotional Support			
Husbands	9.12 (1.21)	9.00 (1.42)	t(1731.36) = -2.02*
Wives	8.65 (1.65)	8.56 (1.86)	t(2156.83) = -1.24
Practical Support			
Husbands	8.58 (1.55)	8.45 (1.71)	t(1784.46) = -1.70
Wives	8.61 (1.55)	8.30 (2.02)	t(2096.8) = -4.001***
Depressive Mood			
Husbands	1.64 (1.81)	1.99 (2.20)	t(659.34) = 2.93*
Wives	2.30 (2.05)	2.42 (2.19)	t(1688) = 1.11
Optimistic Attitudes			
Husbands	7.27 (1.72)	7.02 (1.96)	t(691.50) = -2.25*
Wives	7.24 (1.61)	7.09 (1.77)	t(1686) = -1.76
Perceived Physical Health			
Husbands	1.97 (.61)	2.07 (.64)	t(558.42) = 2.72*
Wives	2.05 (.64)	2.12 (.70)	t(934.54) = 1.92

*Note*: * p < .05,

*** p < .001.

Data source: Swiss Household Panel (SHP).

## Measures

### T1 dyadic support

Emotional and practical support were measured with single-item questions: "To what extent can your partner be available in case of need and show understanding, by talking with you, for example?" (emotional support); "If necessary, in your opinion, to what extent can your partner provide you with practical help, such as concrete help or useful advice?" (practical support). Response options ranged from 0 ("not at all" or “not satisfied at all") to 10 ("a great deal" or "very satisfied"). Scores of the emotional and practical support were averaged with higher scores, indicating greater support.

### T2 mediators

The frequency of depressive mood experienced by the partners was measured through the question: "Do you often have negative feelings such as the ‘blues,’ desperation, or suffer from anxiety or depression?" Response options ranged from 0 ("not at all") to 10 ("always"). The frequency of optimistic attitudes was measured through the question: "Do you often have plenty of strength, energy, and optimism?" Response options ranged from 0 ("never") to 10 ("always”).

### T3 perceived physical health

The perceived health status was measured through the question: "We are now going to talk about various aspects of your health. How do you feel right now?" Response options ranged from 0 ("very well") to 5 ("not well at all"). The score was reversed to obtain an index of physical health.

### Data analysis strategy

T-tests comparing individuals in the initial sample and the analytical sample, and Pearson’s bivariate correlations between variables for distinguishable dyads were carried out on SPSS v. 25. The APIMeM [19] was applied using MEDYAD [18] for SPSS v. 25. MEDYAD is an ordinary least squared (OLS) regression-based approach to mediation analysis with distinguishable dyadic data. We tested two mediators simultaneously (depressive mood and positive attitudes), and we controlled for four covariates (age, educational level, presence of children in the family [dummy-coded], and actual occupation) as potential confounders. The autoregressive effect of health, measured at T2, was also controlled. The errors of the latent variables were correlated to account for couple interdependence.

## Results

Findings revealed degrees of non-independence for all husbands’ and wives’ variables. See Table 2 for detailed results. These results provided grounds to evaluate the couple as the unit of analysis.

**Table 2:** Pearson’s bivariate correlations.

		1	2	3	4	5	6	7
M	8.85	8.63	1.64	2.30	7.27	7.24	4.03	3095
SD	1.21	1.39	1.81	2.05	1.70	1.61	.61	.64
Social support Husbands		.27***	-.13***	-.08*	.17***	0.8*	.06	.06
Social support Wives (1)			-.08*	-.18***	.04	.16***	0.07*	.08**
Depressive mood Husbands (2)				.11***	-43***	-.06	-.22***	-.02
Depressive mood Wives (3)					-.11***	-.49***	-.03	-.28***
Optimistic attitudes Husbands (4)						.09**	.27***	.13***
Optimistic attitudes Wives (5)							.02	.30***
Health Husbands (6)								.09**
Health Wives (7)								

*Note*: * p < .05,

**p < .01,

*** p < .001 two-tailed test.

Data source: Swiss Household Panel (SHP).

**Fig 2:**
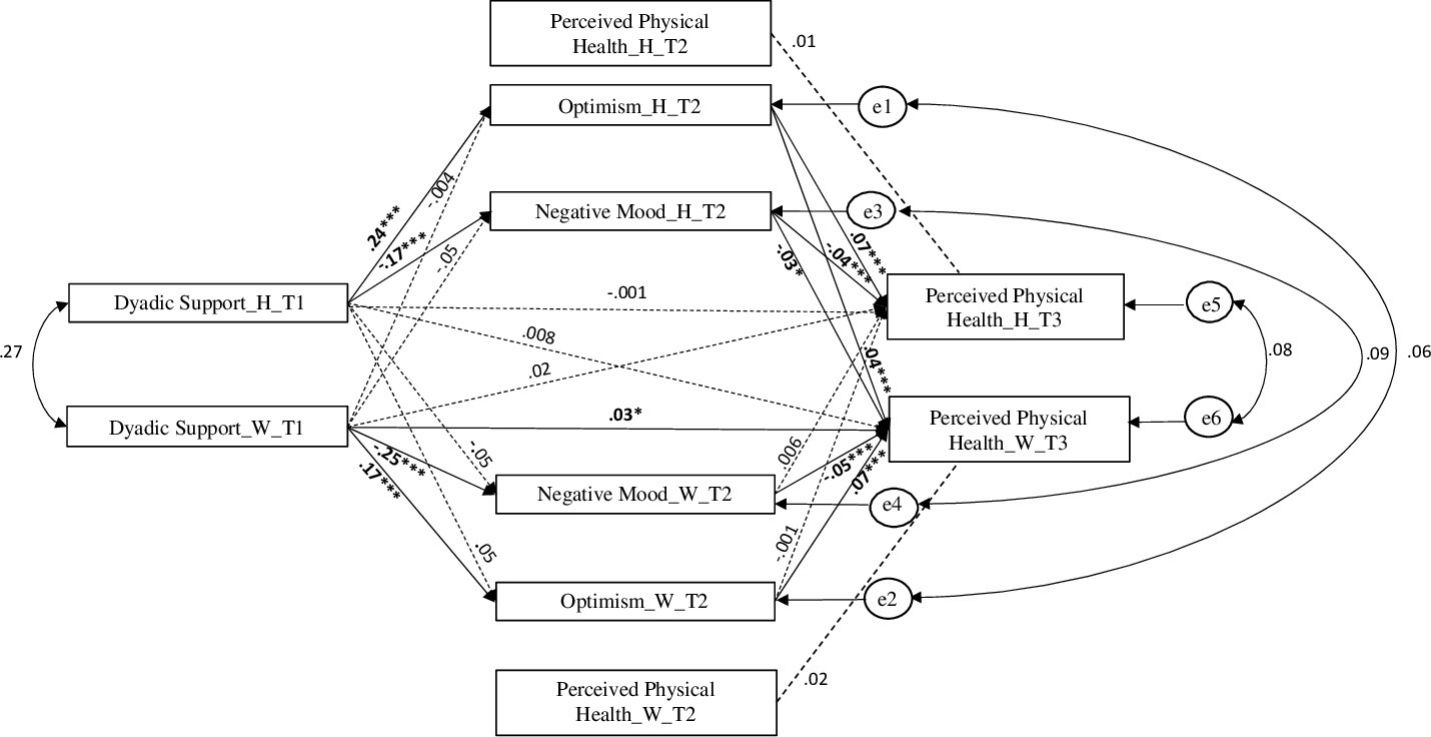
APIMeM with husbands’ and wives’ social support as predictor variables. *Note*: H = husband; W = wife. Plain lines showed the significant paths, dotted lines the non-significant paths. * p < .05, **p < .01, *** p < .001. Data Source: Swiss Household Panel (SHP).

The APIMeM tested the effects of husbands’ and wives’ dyadic support (Fig 2) on their perceived health through the mediation of depressive mood and positive attitudes. The covariates (age, educational level, presence of children in the family [dummy-coded], and actual occupation) and the autoregressive effect of health measured at T2 were not significant.

Results showed significant husbands’ and wives’ actor indirect effects (R^2^_health_husband_ = .31; R^2^_health_wife_ = .37) of the dyadic support on health (see Fig 2). The dyadic support of a partner was significantly associated with improvement of his/her own health (*β* = .07, *p* < .001 for husbands; *β* = .07, *p* < .001 for wives) through the mediation of optimistic attitudes (*β* = .24, *p* < .001 for husbands; *β* = .17, *p* < .001 for wives). Similarly, the dyadic support of a partner was significantly associated with reduction in his/her own depressive mood (*β* = -.17, *p* < .001 for husbands; *β* = -.25, *p* < .001 for wives), and high level of depressive mood was associated with worsening of an individual’s health (*β* = -.04, *p* < .01 for husbands; *β* = -.05, *p* < .001 for wives). A full mediation was found for wives due to the added significant direct effect of dyadic support on perceived health. There were also two other significant partner effects through the mediation of the husbands’ optimistic attitudes. The husbands’ depressive mood reduced their wives’ perceived health (*β* = -.03, *p* < .05), whereas husbands’ optimistic attitudes increased their wives’ perceived health (*β* = .04, *p* < .001).

**Fig 3:**
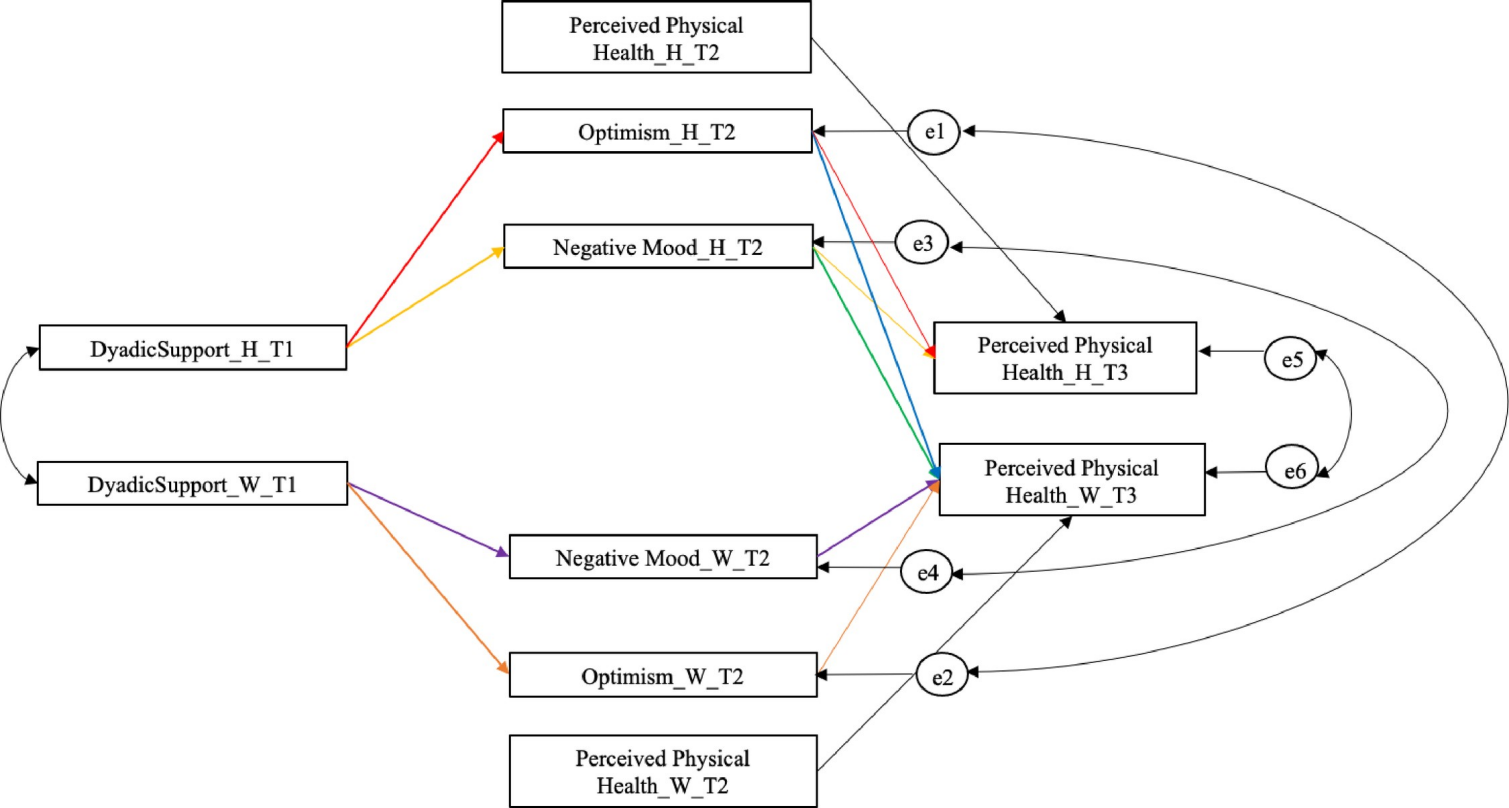
APIMeM with only significant contrasts between indirect actor effects and between indirect actor-partner effects estimated. *Note*: H = husband; W = wife. Dyadic support stands for all the predictor variables (emotional and practical support). Indirect Effect 1 (yellow lines) = Support Husbands →Depressive mood Husbands →Health Husbands; Indirect Effect 2 (red lines) = Support Husbands →Optimism Husbands →Health Husbands; Indirect Effect 3 (purple lines) = Support Wives →Depressive mood Wives→Health Wives; Indirect Effect 4 (orange lines) = Support Wives →Optimism Wives →Health Wives; Indirect Effect 5 (green lines) = Support Husbands →Depressive mood Husbands→ Health Wives; Indirect Effect 6 (blue lines) = Support Husbands →Optimism Husbands→ Health Wives. Data Source: Swiss Household Panel (SHP).

Contrasts comparing significant actor effects and actor/partner effects were calculated (see Table 3 for details). One first contrast (i.e., indirect effect 1 –indirect effect 3) showed that the wife’s perceived dyadic support had a greater effect on her perceived physical health through the mediation of her depressive mood compared to the effect of the similar husband’s actor effect. A second contrast (i.e., indirect effect 2 –indirect effect 4) showed that the husband’s perceived dyadic support had a greater effect on his perceived physical health through the mediation of his optimistic attitudes compared to the effect of the wife’s actor effect. A third contrast (i.e., indirect effect 1 –indirect effect 2) demonstrated that husband’s perceived dyadic support had a greater effect on his perceived physical health through the mediation of his optimistic attitudes compared to the mediation effect of the depressive mood. The fourth contrast (i.e., indirect effect 3 –indirect effect 4) showed a similar trend for wife. See Fig 3 for a graphical representation of the estimated significant contrasts between indirect actor effects and indirect actor-partner effects.

**Table 3:** The contrasts between significant indirect actor effects and between indirect actor-partner effects.

	Effect	BootLLCI	BootULCI
Contrasts between Actor effects			
Indirect Effect 1– Indirect Effect 3	-.0066	-.0162	.0035
Indirect Effect 2—Indirect Effect 4	.0047	-.0077	.0182
Indirect Effect 1—Indirect Effect 2	-.0116	-.0246	.0003
Indirect Effect 3—Indirect Effect 4	-.0003	-.0105	.0096
Contrasts between Actor-Partner effects			
Indirect Effect 5—Indirect Effect 3	-.0176	-.0275	-.0092
Indirect Effect 6—Indirect Effect 4	-.0042	-.0145	.0060

*Note*: - = minus;

Indirect Effect 1 = Support Husbands →Depressive mood Husbands →Health Husbands;

Indirect Effect 2 = Support Husbands →Optimism Husbands →Health Husbands;

Indirect Effect 3 = Support Wives →Depressive mood Wives→Health Wives;

Indirect Effect 4 = Support Wives →Optimism Wives →Health Wives;

Indirect Effect 5 = Support Husbands →Depressive mood Husbands→ Health Wives;

Indirect Effect 6 = Support Husbands →Optimism Husbands→ Health Wives.

Data Source: Swiss Household Panel (SHP).

Alternatively, contrasts between actor and partner effects showed that the actor effects were greater than those of the partner. Particularly, wife’s perceived dyadic support had a greater effect on her perceived physical health through the mediation of her depressive mood compared to the effect exerted by husbands’ perceived support on wife’s health trough the mediation of husbands’ depressive mood. Similarly, wife’s perceived dyadic support had a greater effect on her perceived physical health through the mediation of her optimistic attitude compared to the effect exerted by husbands’ perceived support on wife’s health trough the mediation of husbands’ optimistic attitude.

## Discussion

The current study investigated the effects of the dyadic support on an individual’s health, measured two years later, in a large sample of romantic couples applying the APIMeM design. The actor effects demonstrated the direct association between dyadic support and physical health for wives only and an indirect link to both partners’ mediation of depressive mood and positive attitudes. Our evidence confirmed that the support received by a partner reduced the impact of adverse effects [42] and fostered the effects of positive affective states on health [43]. Consequently, dyadic support received from the partner helps individuals reach a balanced, affective state of mind, sustaining the emotional regulation processes and self-regulatory resources [12] that, in turn, enhance general health. Therefore, an individual’s health is shaped as a phenomenon depending on his/her perception of support received by the partner and on his/her characteristics (e.g., depressive mood and optimistic attitudes).

Several studies have demonstrated the role of specific psychological factors on maintaining good physical and mental well-being such as low levels of depression and negative mood [34, 43, 44] or high levels of positive attitudes [52] such as dispositional optimism [45–48]. In particular, both the meta-analytic review by Rasmussen and colleagues [48] and the review by Carver and Scheier [45] suggested that dispositional optimism promotes physical health while dispositional pessimism, measured as depressive mood, impaired physical health. People with high optimistic attitudes or low depressive feelings showed decreased levels of inflammation and cortisol, increased antioxidant levels, and were less likely to develop chronic diseases than people with low optimism or high depression. Optimists seem to take a proactive approach to health promotion [45]. Optimistic individuals also adopt a better profile of emotional responses and coping mechanisms for stressful events in terms of a low level of distress and high levels of positive emotions [47]. Accordingly, many studies have explained that optimism is related to less perceived stress and mood disturbance in response to stressful events [53, 54] as well as in the absence of specific stressors [46, 52].

Regarding partner effects, the wives’ perceived health was predicted by the husbands’ support through the mediation of the husbands’ optimistic attitudes. The husbands’ perceived health affected their wives’ dyadic support through the mediation of the husbands’ depressive mood. These findings are related to an individual’s ability to influence another person’s behaviors, emotions, and thoughts due to the interconnection between the recipient, the provider, and their relationships [4]. The research demonstrated that men experience more significant health benefits through the positive lifestyle and health behaviors from a stable relationship with their wives [55], and they experience lower costs from spousal caregiving, childrearing, caring for aging parents, and balancing work/family demands [56]. Although historically, men have been considered to benefit more from marriage than women, our results demonstrated that the positive quality attributed to a stable relationship is valid also for women. According to several studies [13, 37–41], both personal (attitudes and mood) and relationship (dyadic support) factors allow romantic partners to cope with everyday life events. Specifically, they permit improving individuals’ perceived health through the mediation of positive mood, which is the low depressive mood and high dispositional optimism, as previously demonstrated [46, 48].

The dyadic support is co-constructed by each member of a couple through constructive dyadic communication processes. Dyadic support between romantic partners is derived from everyday exchanges about their worries, feelings, or needs [4]. In romantic relationships, each member has a concern for the partner’s welfare and a very high degree of motivation to respond to the other’s need [57–61]. This field of Relationship Science [62] has shown that the quality of the relationships should be considered more important than the number of relationships because the high quality of close relationships with others is essential for human well-being.

One bias that affects the psychological research in this field is the application of adequate models and statistical tools that assess interpersonal processes [22]. The present study confirmed the benefits of interpersonal factors, such as dyadic support, on health [13, 63] by applying a dyadic design. It also demonstrated the interplay between an individual’s attitudes and mood as well relationship factors, which contribute to better individual health.

Another bias in this field is the fact that there is a general tendency to focus on specific health stressors, also referred to as ‘we-diseases’ (i.e., life-threatening diseases such as cancers) [64], because of their impact on the individual’s health and the couple’s well-being and quality of life. Our study strength is the implementation of those effects during the couple’s everyday life in the absence of such adversities. Stable relationships allow for the implementation of coping strategies that regulate and maintain the couple’s homeostasis, therefore influencing and reinforcing the relationship itself.

These mechanisms underline the importance of considering the couple as the unit of analysis when focusing on health-related behavioral tendencies, such as diet, physical exercises, alcohol consumption, or smoking habits. Such dyadic processes should be investigated when a health behavior change is needed, even in the absence of disease. Our evidence should be extended, focusing on support, dyadic coping, and other dynamics in couples to investigate how they interact in determining behavioral health tendencies.

Moreover, further studies should apply dyadic data models like the APIMeM to other interpersonal communication processes between partners and investigate, for example, whether the partners’ other variables (e.g., stress, coping strategies) would influence one’s own perceived health status. It also would be interesting to distinguish the dyadic support in terms of support received and provided (and the delta between them) and measure the effect on health.

## Limitations

Despite the advanced methodology applied in the present research and the longitudinal design, some limitations occurred. First, the Swiss Household Panel did not collect the considered variables every year; therefore, we cannot evaluate the auto-regressive effects or the interrelations among variables. Future research should measure dyadic support, depressive mood, optimistic attitudes, and health across the years, estimating their interrelations with a cross-lagged model. Second, we considered only the perceived health status as an outcome, whereas future research may distinguish between physical health and mental health. Third, the variables were measured through single-item questions, which is a limitation for the validity of the scale and might be biased by social desirability. Finally, although the sample size in this study was pretty high, considering that we analyzed dyadic data, it was not possible to test the model on the original full SHP sample because of the missing data completely at random. Further studies should re-test this model considering a representative sample of the population.

## Conclusions

In conclusion, the present study demonstrates the benefits of social and personal factors on maintaining good health. Indeed, the most critical result concerns the effect of one partner’s perception of the dyadic support on the other’s health. It would be useful to understand the specific mechanisms within close relationships, even in daily life, to improve and develop intervention guidelines.
